# Bio-medical entity prioritisation based on literature with Semantic Web annotations

**DOI:** 10.1186/1753-6561-9-S5-A6

**Published:** 2015-08-06

**Authors:** Norio Kobayashi

**Affiliations:** 1 Advanced Center for Computing and Communication (ACCC), RIKEN, Wako, Japan

## Summary

An extension of the General and Rapid Association Study Engine (GRASE), a Semantic Web data (entity) prioritisation engine, is discussed. The GRASE employs a unique mechanism to prioritise entities using entity-document relations by computing the statistical significance between entities and user keywords based on the number of related documents. We describe an improvement of prioritisation accuracy and connectivity to the Semantic Web using PubAnnotation.

## Introduction

In life-science data analysis, prioritisation of entities among a large number of candidates is an important task. The General and Rapid Association Study Engine (GRASE) [1] introduced here is a Semantic Web data prioritisation engine. The GRASE was originally used for causative gene search in a RIKEN mouse ENU-mutagenesis program and 65 genes were successfully highly ranked [2] through its web interface called PosMed [3]. The supported data has been extended and used successfully to discover bioresources in mice and *Arabidopsis*. The unique characteristic of GRASE prioritisation is employing entity-document relations where documents include MEDLINE abstracts. This mechanism has an advantage in which each prioritised entity can be shown with related documents as evidence, and these entities can be searched even if their descriptions are not given as Semantic Web data. However, the precision of such functions depends strongly on the accuracy of the entity-document relations. In the following, we examine the GRASE with a PosMed example and propose an extension using PubAnnotation [4] to improve accuracy and provide further support for Semantic Web data.

## Statistical prioritisation of GRASE

The core functions of the GRASE are (1) keyword-entity search and (2) entity-entity search, which includes both (2.a) literature co-citation search and (2.b) semantic link search.

**Figure 1:**
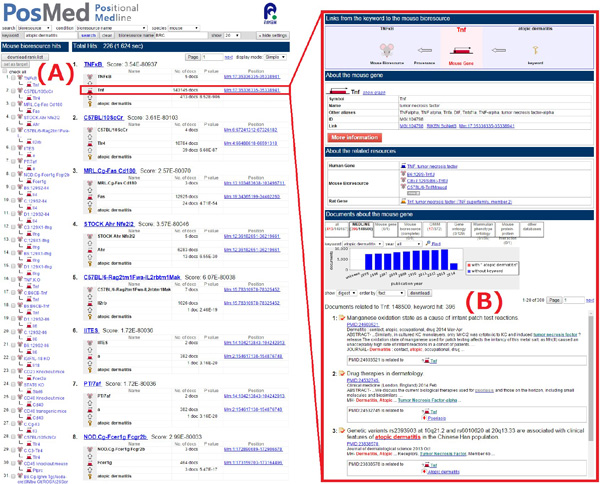
**PosMed search example of ranked mouse bioresources with keyword 'atopic dermatitis'**. (A) Resultant ranking of bioresources; (B) list of documents (including MEDLINE abstracts and OMIM) associated with the mouse genome informatics (MGI)-mouse gene [6] Tnf connected by a semantic link to bioresource TNF*κ*B (ranked first). Documents associated with the user keyword, terms associated with TNF*κ*B and terms associated with other entities are highlighted in red, green and grey, respectively.

Here, we view the GRASE prioritisation process with an example that ranks the bioresources of mice [2,5] by specifying the keyword 'atopic dermatitis' using search path *keyword (1)→ mouse gene (2.b)→ mouse bioresource *in PosMed (Figure 1).

First, (1) keyword-entity search, denoted *keyword (1)→ entity*, is executed for each mouse gene. The keyword-entity search is performed to rank entities by computing the statistical significance of associations between a user keyword and each entity using entity-document relations. For each instance, a 2 × 2 contingency table, i.e.

$$\left(\begin{array}{cc} a & b\\ c & d \end{array}\right),$$

consisting of the number of documents where (a) both the entity and the keyword appear, (b) the keyword appears but the entity does not, (c) the entity appears but the keyword does not and (d) neither the entity nor the keyword appear. Then, Fisher's exact test is applied to the contingency table, and the *P*-value is computed as the statistical significance.

Next, (2.b) entity-entity search based on the semantic links, denoted *entity (2.b)→ entity*, is performed on the result of (1). For each result of (1), the GRASE discovers mouse bioresources linked from the resultant mouse gene and its *P*-value is given as 0. Therefore, the total *P*-value of the search path is the *P*-value of (1). Finally, mouse bioresources are ranked by the *P*-value of their paths.

## Association between literature and entities

In the current implementation, entity-document relations are obtained by full-text search with human-curated logical queries defined as a list of entity names concatenated with logical operators such as **AND**, **OR **and **NOT**. For example, the logical query for the *Arabidopsis *gene AT1G03880 (cruciferin B, CRB) is defined as follows:

(AT1G03880 OR CRU2 OR CRB OR 'CRUCIFERIN 2' OR 'CRUCIFERIN B')

AND (Arabidopsis) NOT ('chloroplast RNA binding').

Logical queries for mouse, human and *Arabidopsis *genes have already been curated manually.

## Discussion

Here, we discuss GRASE statistical prioritisation search, which ranks entities using entity-document relations. Although our curation method to obtain entity-document relations is effective for frequently updated arbitrary document sets, it may still produce false-positive errors and cannot be extended for additional entities. The open collections of annotations of literature, including PubAnnotation can be used to solve this problem, which allows GRASE search to be performed more precisely. Another advantage of introducing PubAnnotation is the ability to show documents with detailed annotation with semantic links.

Therefore, future work includes implementing an interface to input/output PubAnnotation data. More concretely, we would like to introduce PubAnnotation data as accurate entity-document relations in addition to our query approach and implement functions to display and download documents that are related to the resultant entity in PubAnnotation format.
